# Allogenous Selection of Mutational Collateral Resistance: Old Drugs Select for New Resistance Within Antibiotic Families

**DOI:** 10.3389/fmicb.2021.757833

**Published:** 2021-10-22

**Authors:** Fernando Baquero, José L. Martínez, Ângela Novais, Jerónimo Rodríguez-Beltrán, Laura Martínez-García, Teresa M. Coque, Juan Carlos Galán

**Affiliations:** ^1^Department of Microbiology, Ramón y Cajal University Hospital, Ramón y Cajal Institute for Health Research (IRYCIS), Network Center for Research in Epidemiology and Public Health (CIBERESP), Madrid, Spain; ^2^Department of Microbial Biotechnology, National Center for Biotechnology (CNB-CSIC), Madrid, Spain; ^3^UCIBIO – Applied Molecular Biosciences Unit, Laboratory of Microbiology, Department of Biological Sciences, REQUIMTE, Faculty of Pharmacy, University of Porto, Porto, Portugal; ^4^Associate Laboratory i4HB - Institute for Health and Bioeconomy, Faculty of Pharmacy, University of Porto, Porto, Portugal

**Keywords:** allogenous selection, synergistic pleiotropy, collateral hyper-resistance, collateral selection, collateral expansion, collateral evolution, antibiotic resistance mutations

## Abstract

Allogeneous selection occurs when an antibiotic selects for resistance to more advanced members of the same family. The mechanisms of allogenous selection are (a) collateral expansion, when the antibiotic expands the gene and gene-containing bacterial populations favoring the emergence of other mutations, inactivating the more advanced antibiotics; (b) collateral selection, when the old antibiotic selects its own resistance but also resistance to more modern drugs; (c) collateral hyper-resistance, when resistance to the old antibiotic selects in higher degree for populations resistant to other antibiotics of the family than to itself; and (d) collateral evolution, when the simultaneous or sequential use of antibiotics of the same family selects for new mutational combinations with novel phenotypes in this family, generally with higher activity (higher inactivation of the antibiotic substrates) or broader spectrum (more antibiotics of the family are inactivated). Note that in some cases, collateral selection derives from collateral evolution. In this article, examples of allogenous selection are provided for the major families of antibiotics. Improvements in minimal inhibitory concentrations with the newest drugs do not necessarily exclude “old” antibiotics of the same family of retaining some selective power for resistance to the newest agents. If this were true, the use of older members of the same drug family would facilitate the emergence of mutational resistance to the younger drugs of the family, which is frequently based on previously established resistance traits. The extensive use of old drugs (particularly in low-income countries and in farming) might be significant for the emergence and selection of resistance to the novel members of the family, becoming a growing source of variation and selection of resistance to the whole family. In terms of future research, it could be advisable to focus antimicrobial drug discovery more on the identification of new targets and new (unique) classes of antimicrobial agents, than on the perpetual chemical exploitation of classic existing ones.

## Introduction

Within a given family of antibiotics, the acquisition of mutational resistance in the chromosomal target or in transport genes to one of the antibiotic members frequently involves resistance to many or all other members of the family (cross-resistance). The emergence of resistance is an “antibiotic-specific” phenomenon, i.e., the use of antibiotic “*a1*” of the family “*A*” preferentially favors the selection of mutations providing resistance to “*a1*” and not necessarily (or less effectively) to “*a2*.” This is explained by the frequent commonality of pharmacodynamic parameters. In the case of mutations in target or antibiotic-modifying (inactivating) genes, selection with “*a1*” favors the mutational emergence of “*a1*” resistance and to a lesser degree to other members. For example, within a single family, aminopenicillins (such as ampicillin or amoxicillin) select for aminopenicillin resistance; or oxyimino-cephalosporin selects for resistance to this group of antibiotics, such as cefotaxime (CTX) or ceftazidime (CAZ). However, this is not always true. We will discuss this issue, analyzing the cases in which the use of an *a1* antibiotic favors the emergence and spread of resistance to an *a2* antibiotic, leading to “allogeneous selection.”

The phenomenon of “collateral-susceptibility” refers to the case in which resistance to a particular antibiotic is associated with high susceptibility to another antibiotic. Collateral susceptibility not only occurs (as is commonly believed) among antibiotics of different groups (Podnecky et al., 2018), but also among members of the same family. Mutational resistance to an A member occasionally increases the susceptibility to other “A” family drugs, indicating an asymmetry of the phenotypes resulting from mutational events involved in the acquisition of resistance (Sanz-García et al., 2019). This asymmetry is the main conceptual point making allogeneous selection a particularly interesting aspect of the classic “selection by cross-resistance.” In fact, pleiotropy occurs when a single mutation affects multiple phenotypic traits. In other words, allogenous selection occurs when a given antibiotic selects mutations that confer resistance to another, often more potent or advanced, member of the same antibiotic family, either directly or by enriching the frequency of mutations at the population level that facilitate the emergence of resistance to the second antibiotic.

## Four Cases Determining Allogenous Selection

**Figure 1:**
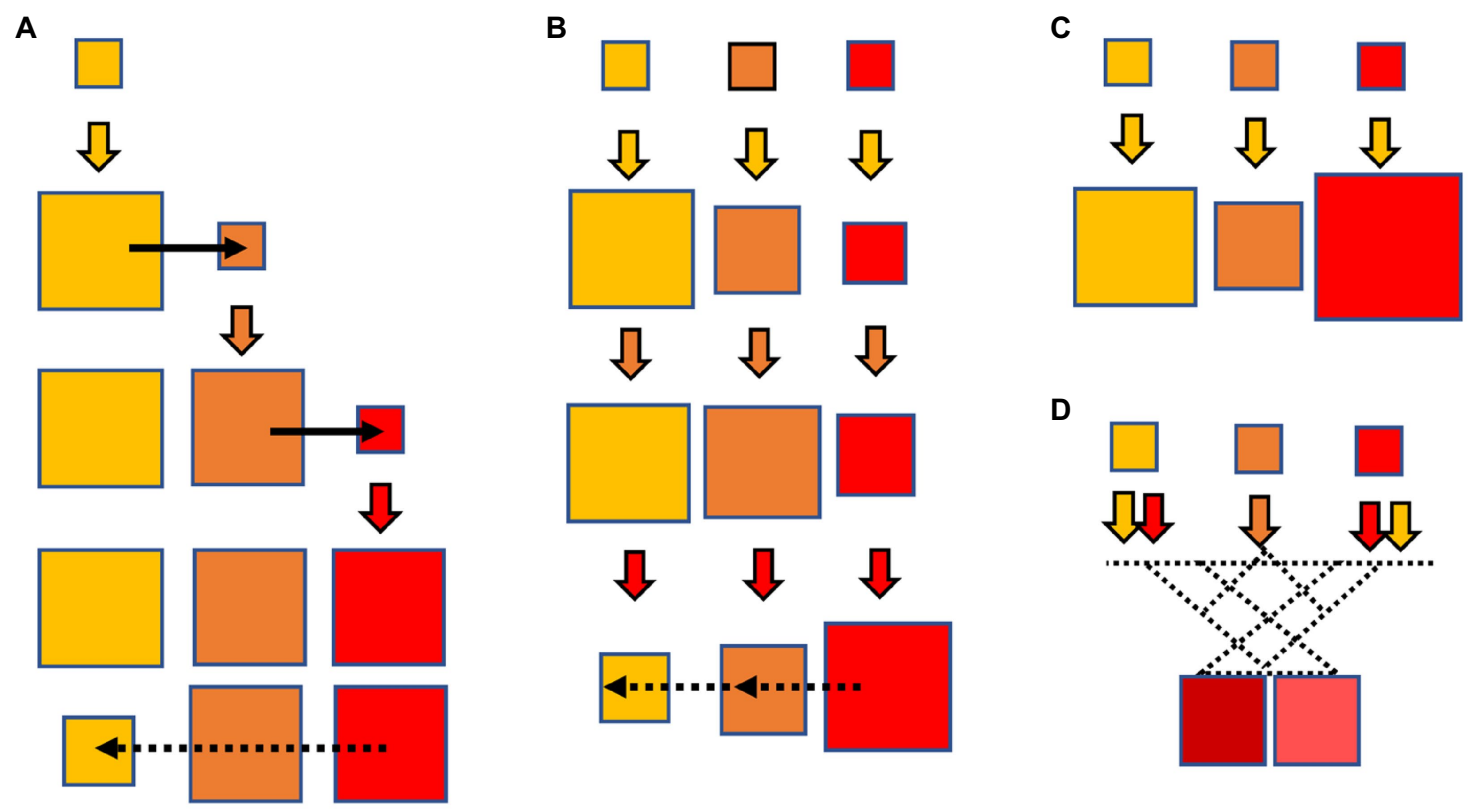
Four cases of allogeneous selection, in which the use of an antibiotic (solid-colored arrows, the potency of the antibiotic increasing in order from yellow to brown to red) results in the emergence of mutants with resistance to other antibiotics of the same family, even the more advanced ones. **(A)** Collateral expansion: the yellow antibiotic expands the gene and species populations with mutational resistance to the yellow drug (yellow square), favoring the emergence of other mutations (black arrow) and inactivating the brown antibiotic; the brown antibiotic expands these populations, favoring new mutations producing resistance to the red antibiotic, which are selected by this drug. Note that this final effect starts with use of the yellow (less potent) antibiotic, and results in resistance to the red one (more potent). Eventually, resistance to the red antibiotic might reduce the efficiency of resistance to the yellow one, by antagonistic pleiotropy or collateral susceptibility (dotted black arrows). **(B)** Collateral selection: the yellow antibiotic selects the populations with yellow resistance but also (to a minor extent) the brown and red resistance. However, further exposure to brown and red drugs selects for these resistant populations, but the trigger of the process is the weaker antibiotic; again, collateral susceptibility might occur (dotted black arrows). **(C)** Collateral hyper-resistance: the yellow antibiotic selects for populations resistant to other antibiotics to a higher degree than to itself. **(D)** Collateral evolution: when the simultaneous or sequential use of antibiotics of the same family selects for new mutational combinations with novel phenotypes, generally with higher activity or broader spectrum. In some cases, collateral selection derives from collateral evolution.

In this review, we consider several cases determining allogenous selection, as illustrated in Figure 1. First, the case of “collateral expansion” (Figure 1A), in which the use of a particular antibiotic increases the absolute frequency of a resistance determinant, which results in a greater chance of evolution to increased resistance to other novel member(s). For example, the use of ampicillin leads to enrichment of *bla* genes such as *bla*_TEM-1_ or *bla*_SHV-1_. The expansion of these *bla* genes could facilitate the mutational emergence of evolved variants called extended-spectrum β-lactamases (ESBLs). This emergence facilitates the mutational emergence of TEM-1- or SHV-1- derived ESBLs, inactivating oxyimino-cephalosporins and resulting in an allogenous selection of these novel drugs. Second, the case of “collateral selection” (Figure 1B), in which allogenous selection occurs between subgroups of antibiotics of the same family. For example, the use of ampicillin or cefalexin (a first-generation cephalosporin) should selects for resistance to third-generation cephalosporins, and viceversa (Quinn et al., 1989). That is even more evident for CTX-M enzymes, readily hydrolyzing aminopenicillins and first generation cephalosporins (Ghiglione et al., 2018), except for CTX-M-93 (Djamdjian et al., 2011). In animal colonization models, ampicillin selects for CTX-M harboring bacteria just a little less effectively than ceftazidime (Stercz et al., 2021). Third, the case of “collateral hyper-resistance” (Figure 1C) within different families of antibiotics, in which exposure to an antibiotic “*a1*” favors the emergence of mutational resistance, resulting in a higher mean minimal inhibitory concentration (MIC) of another antibiotic of the same family “*a_n_*” absent in the selective process. For example, ceftazidime selects for VIM genetic variant strains with high carbapenemase activity more efficiently than carbapenems themselves. Fourth, the case of “collateral evolution” (Figure 1D), in which the combined effect of 2 or more antibiotics from the same family favors the emergence and further evolution and selection of novel resistance phenotypes, eventually more efficient to inactivate the ensemble of causative drugs.

## Allogeneous Selection Within Β-Lactams

Collateral expansion has strongly influenced the spread of antibiotic resistance within β-lactams. The emergence of TEM-1- or SHV-1-derived ESBLs, hydrolyzing oxyimino cephalosporins, has occurred because of the wealth (absolute abundance) of *bla*_TEM-1_, *bla*_TEM-2_ or *bla*_SHV-1_ genes resulting from the overprescription of aminopenicillins. β-lactamases are enzymes with a high tolerance to amino acid changes (Zaccolo and Gherardi, 1999), providing a highly plastic genetic background for mutational variation. The antibiotic-driven abundance of TEM-1 and SHV-1 enzymes favored the selection of mutational events when cefotaxime (CTX) became available and was widely used. Selection for CTX resistance in TEM-1-harboring strains is accompanied by smaller increases in CAZ resistance than vice versa (Schenk et al., 2015). Thus, selection by aminopenicillins has contributed to ESBL evolution. It will be of interest to search for TEM-derived ESBLs before 1983, the year when cefotaxime was launched. The number of natural TEM enzymes is much larger and could have led to cefotaxime-resistant variants that originated in TEM-1 or TEM-2, which are just one part of a complex network of TEM enzymes with related sequences (Zeil et al., 2016). In addition, there is collateral susceptibility to aminopenicillins in strains harboring ESBLs such that alleles inactivating CTX become more susceptible to these drugs, a phenomenon of antagonistic pleiotropy. However, it can be suspected that the continued use of aminopenicillins could contribute to the selection of ESBLs. In fact, the ampicillin specificity constant, *kcat/Km*, which allows a comparison of the activity of various β-lactamases on this substrate, is only 40 times lower for the ESBLs TEM-5 and TEM-10 than for TEM-2 (Quinn et al., 1989). In the case of ESBL derivatives of SHV-1 harboring the changes G238S or G238A, the difference is even lower, only between 6 and 3 times (Hujer et al., 2002). That difference suggests to us and others (Karen Bush, personal communication) that aminopenicillins might be sufficiently hydrolyzed by ESBLs to select for ESBL-containing organisms. However, most ESBL-containing organisms retain early aminopenicillin-hydrolyzing TEM or SHV enzymes even in the presence of ESBLs, which probably minimizes specific selection of ESBL-only strains, as has been indicated by membrane-computational models (Campos et al., 2019). In any case, the frequent coexistence of genes encoding early TEM-1 or SHV-1 β-lactamases with ESBLs ensures that strains with ESBLs are also selected with aminopenicillins or first-generation cephalosporins. Certainly, first-generation cephalosporins contribute to the emergence and selection of *in vivo* resistance to CTX (Kimura et al., 2017). Even ampicillin can select for metallo-β-lactamases such as NDM-1, which inactivate aminopenicillins (Zhang and Hao, 2011).

Another example of collateral expansion leading to allogenous selection within β-lactams is the evolution of mutations in *Streptococcus. pneumoniae* penicillin-binding proteins (PBPs) leading to resistance to third-generation cephalosporins. The use of penicillins from the 1940s, and particularly the overuse of aminopenicillins from the 1960s, has selected *S. pneumoniae* low-level resistant strains. This also occurs with *in vitro* selection, yielding mutants in PBP2x and PBP2b: strains with higher levels of penicillin resistance occur because of added cumulative changes in PBP1a. PBP2x mutants are also involved in CTX resistance (Maurer et al., 2008), and penicillin-triggered multiple mutants, including those in PBP1a, significantly increase resistance to CTX (Smith et al., 2001; Schrag et al., 2004). In summary, the use of penicillins expanded the PBPs’ genetic substrates, leading to the selection of CTX resistance. Finally, the emergence of the SCCmec element, giving rise to methicillin-resistant *Staphylococcus aureus* was also probably associated with the use of penicillins in the 1940s and 1950s and not necessarily with exposure to methicillin-oxacillin, launched 15years later (Harkins et al., 2017).

Collateral hyper-resistance (Figure 1C) occurs with β-lactams. Although it seems obvious that the ability to hydrolyze carbapenems would have resulted from an increasing exposure to carbapenems, several studies have demonstrated that oxyimino-cephalosporins are more efficient in this evolutionary process. Using VIM enzymes as a model, we demonstrated that CAZ, better than CTX or any carbapenem (imipenem, meropenem or ertapenem) was most probably responsible for the evolution of variants toward more efficient carbapenem hydrolysis (Galán et al., 2013; Martínez-García et al., 2018). The same can be observed in experimental evolution studies on KPC-2 carbapenemase, in which evolved single and double mutational variants showed a higher catalytic efficiency toward CAZ than toward carbapenems (Mehta et al., 2015).

It has also been observed that the exposure to ceftazidime favors certain mutational events in CTX-M enzymes that increase MIC to CAZ but with very different effects depending on the aminoacid position(s) involved. Positive selection on specific amino acid mutation sites is required to obtain high fitness peaks toward efficient CAZ hydrolysis, but also secondary mutations are needed to guarantee a more balanced CTX-CAZ resistance phenotype, whereas other β-lactams, such as cefuroxime, are much less affected (Novais et al., 2008). This observation uncovers the role of the combinatorial exposure to different β-lactams (cefuroxime, CTX, CAZ, and/or cefepime) on CTX-M collateral evolution, as described below.

Collateral evolution is also observed within β-lactam resistance determinants. The simultaneous or sequential exposure to β-lactams contributes to the emergence and selection of new β-lactamases through various combinatorial mutational trajectories, giving rise to a plethora of intermediates from which evolution can occur. From multiple possible evolutionary trajectories, the emergence of the most evolved and efficient variants (e.g., CTX-M-32, CTX-M-58) as well as several CTX-M-3 derivatives already described, can be easily explained by a fluctuating exposure to both cefotaxime and ceftazidime that would favor some of these trajectories (Novais et al., 2010). This would favor an increase in frequency of a series of mutational variants and their subsequent evolution toward a balanced hydrolysis of both CTX and CAZ, which constitutes an example of collateral evolution.

From the diverse possible evolutionary paths in CTX-M-1 gene, some were predicted to occur more frequently, such as those including the mutation D240G, precisely because they more frequently generate variants that consistently confer an increased catalytic efficiency to both oxyimino-cephalosporins, CTX and CAZ. In fact, data from the β-lactamase database as of July 2021^1^ confirm these predictions. Given almost half (*n*=102/213; 48%) of the CTX-M-described variants contain the D240G mutation (Novais et al., 2010). This study showed that P167S variants are those that confer higher catalytic efficiency to these drugs. Experimental evolution assays based on serial passages under increased concentrations of an oxyimino-cephalosporins frequently yielded mutants that did not correspond to those more commonly detected in nature (Novais et al., 2008). This result might indicate that the most successful long-term strategy follows slower trajectories, such as the D240G mutation, a phenomenon known as the “survival of the flattest” (Day et al., 2015). The fittest organisms in terms of MIC (selected under high antibiotic concentrations) could be less robust than those with lower increases in enzymatic activity.

It is also reasonable to hypothesize that the exposure of a wealth of CTX-M variants (with favorable phenotypes to CTX, CAZ or both) to β-lactam/β-lactamase inhibitor combinations would also extend the spectrum of these β-lactamases. As another example of collateral evolution giving rise to combined phenotypes, specific mutations (S237G and K234R) on specific genetic backgrounds are able to produce slight increases in MIC to either amoxicillin-clavulanate or piperacillin-tazobactam combinations while maintaining the efficiency toward cephalosporins (Ripoll et al., 2011). However, these MIC levels are still in the susceptibility range and might go unnoticed by routine procedures or detected only when combined with additional mechanisms (e.g., production of OXA-1, outer membrane permeability defects, or insertion sequences upstream of an antibiotic-resistance gene; Ripoll et al., 2011). However, this situation is not very common, given the same or other mutations in variable CTX-M backgrounds drastically reduce the MIC to second- and third-generation cephalosporins (Ripoll et al., 2011; Rosenkilde et al., 2019). This collateral sensitivity effect could constrain the evolution of variants toward these combined phenotypes, which is why certain combinations (cephalosporins combined with β-lactam inhibitors) could be explored to counteract/block evolution of antibiotic resistance. In fact, *in vitro*, the combination of CTX and mecillinam prevented CTX-M-15 to evolve toward combined resistance to other β-lactams compared with each of these antibiotics alone (Rosenkilde et al., 2019).

We also need to consider that some of these conclusions arise from a small number of experimental replicates and that collateral responses might be more diverse. Experimental evolution assays with mathematical modelling assessing SHV-1 evolutionary trends under CTX exposure have shown not only a divergent collateral response but also that sensitivity to a second drug depends on the type of first mutation that arises (Nichol et al., 2019). By simulating diverse sequential combinations of β-lactams, the authors also showed that cross-resistance, and thus collateral selection, frequently occur to cefazolin and ceftolozane-tazobactam. The cross-resistance reported between ampicillin (first drug) and CTX (second drug) suggests also that ampicillin exposure could have favored diversification of SHV-1 and selection of mutants with increased advantage over CTX, contributing partly to the selection of *Klebsiella pneumoniae* strains.

Multidrug efflux pumps contribute to intrinsic resistance to several antibiotics. These pumps also contribute to an MIC increase to several β-lactams when they are overexpressed. In recent years, it has been shown that mutants in the structural elements of efflux pumps might modify their substrate profile. A good example of this is mutations in SmeH, a resistance-nodulation-division efflux pump transporter from *Stenotrophomonas maltophilia*, resulting in small increases in MICs to cefazolin (a first and cephalosporin), cefoxitin, and CTX. These mutations can certainly be selected by these antibiotics, but the result is a significant increase in resistance to more “advanced” antibiotics such as CAZ and occasionally aztreonam (Blanco et al., 2019).

## Allogeneous Selection Within Macrolides

Most cases of allogenous selection of macrolide-resistant mutants involving other macrolide-lincosamide-streptogramin B (MLS) antibiotics correspond to complete (all MLS) or incomplete symmetrical cross-resistance, allogenous collateral selection. In any case, the selection of strains with mutational resistance to old drugs can lead to increases in resistance to some of the novel ones. In fact, mutational resistance to old drugs (such as erythromycin) might produce increases in MICs to other MLS drugs, but this is not always the case (particularly with the newest macrolides), and infrequently the MIC increase for other drugs surpasses the MIC increase for erythromycin. However, allogenous hyper-resistance was found in azithromycin-resistant *Streptococcus pyogenes* mutants, increasing spiramycin resistance to a higher degree than that of azithromycin itself (Malbruny et al., 2002). Several mutations in the ribosomal macrolide binding site L4/L22 proteins and the 23S rRNA domains II/V selected under long-term serial erythromycin exposure in *Staphylococcus aureus* increased the MICs to ketolides (telithromycin or solithromycin), and lincosamides but not greater than the level of erythromycin resistance (Yao et al., 2018). Mutations in the 23S rRNA gene (such as A2142G) of *Helicobacter pylori* are generally associated with cross-resistance to all MLS antibiotics, but not in all cases: erythromycin-resistant strains might maintain susceptibility to clarithromycin (Wang and Taylor, 1998; García-Arata et al., 1999). In *S. pneumoniae*, erythromycin-resistant mutations obtained by serial passages are expected to contribute to selection of the resistant strain by exposure to 15- and 16-membered macrolides, streptogramin, and less frequently to lincosamides, but not to telithromycin (Tait-Kamradt et al., 2000), and this trend is expected to occur with other organisms.

Macrolides are common substrates of multidrug efflux pumps. The study of clinical *Haemophilus influenzae* isolates has shown that various mutations in the AcrR regulator, which triggers AcrAB pump expression, slightly increase azithromycin susceptibility without reaching breakpoints defining resistance and renders clarithromycin resistance (Seyama et al., 2017). These cases suggest that the use of azithromycin might have fewer consequences on azithromycin resistance than on resistance to the allogenous agent, clarithromycin, indicating a possible case of allogenous hyper-resistance.

## Allogeneous Selection Within Tetracyclines

*Escherichia coli* resistance determinants encoding tet(M), tet(K), tet(A), and tet(X) detoxify first-generation tetracycline (high MICs) but increase MICs to second-generation drugs such as doxycycline and minocycline, and also to those of the third generation, tigecycline and eravacycline (Grossman, 2016). Tigecycline and eravacycline MICs remain unchanged in strains with tet(B). The tet(X) resistance protein, a flavin-dependent monooxygenase, inactivates first- and second-generation tetracyclines (Moore et al., 2005), but some of its mutational variants increase minocycline or tigecycline MICs more than tetracycline itself (Cui et al., 2021). As in the case of classic antagonistic pleiotropy β-lactamases evolving to ESBLs or inhibitor-resistant TEMs, *tet*(M) mutants selected for increased tigecycline MICs might lose resistance against earlier tetracycline antibiotics (Linkevicius et al., 2015). Accordingly, with the Imamovic and Sommer map of “collateral sensitivity,” in which a mutant to one antibiotic results in increased susceptibility to another (Imamovic and Sommer, 2013), minocycline-resistant mutants might increase resistance to tigecycline to a greater extent than to minocycline (its predecessor molecule) itself.

Undoubtedly, the main allogeneic selection by tetracyclines is the expansion of tetracycline resistance determinants, fostering the emergence of genetic variants that increase tetracycline resistance, which also influences (inactivates) the newest members of the family. These genetic variants probably include *tet* mosaic genes yielding higher MICs to tetracycline, such as *tet*(O)-*tet*(W) mosaic genes (Stanton et al., 2004).

## Allogeneous Selection in Quinolones-Fluoroquinolones

Collateral expansion of quinolone-fluroquinolone resistance is evident. The emergence of fluoroquinolone resistance followed the previous selection (enrichment) of quinolone resistance genes by first quinolone-based drugs such as nalidixic acid, oxolinic acid, cinoxacin, and pipemidic acid, widely used in the community in the 1960s and 1970s (Emmerson and Jones, 2003; Ruiz, 2019). These quinolones provided selective power for resistant variants harboring a significant first mutational step, paving the way toward fluoroquinolone resistance by acquisition of a second mutation. Therefore, at the time of launching norfloxacin in 1986, this enriched genetic background was ready for the unexpected rapid emergence and spread of fluoroquinolone resistance (Aguiar et al., 1992). One striking example can be that of *qnrB* alleles described in isolates from as early as in the 1930’s (Saga et al., 2013) well before quinolones’ market introduction. Their diversification within *Citrobacter* spp. and other *Enterobacteriaceae* after horizontal gene transfer mobilization explains their high allele diversity and current abundance (Ribeiro et al., 2015). Many of these variants have stable nalidixic acid phenotypes (Rodríguez-Martínez et al., 2009) and could eventually be selected by first generation quinolones, increasing the opportunity for resistance to newer generation quinolones to arise.

Collateral hyper-resistance might occur within quinolones-fluoroquinolones. Selection of double mutations in topoisomerases by ciprofloxacin (typically involving positions 83 and 87 of *gyrA*) probably increases the MIC of nalidixic acid five times more than the original ciprofloxacin MIC (Vila et al., 1994). Thus, the continued use of quinolones (such as nalidixic acid, oxolinic acid, cinoxacin and pipemidic acid) in the 1960s and 1970s should have facilitated an efficient selection of fluoroquinolone resistance, introduced in the late 1980s or later, such as norfloxacin, ciprofloxacin, ofloxacin, moxifloxacin, tosufloxacin, or sitafloxacin (Honda et al., 2020). Nalidixic acid is used in South-East Asian countries mainly to treat *Shigella* infections (Hoge et al., 1995). Today, nalidixic acid is still consistently found in European rivers (Castrignanò et al., 2020), and pipemidic acid is consistently present in European wastewater treatment plants (Rodriguez-Mozaz et al., 2020), and possibly will continue to serve as selectors for resistance to the newest fluoroquinolones. Similarly, exposure to fluoroquinolones such as ciprofloxacin and ofloxacin typically selects mutants that show increased resistance to these drugs, but even higher levels of resistance to more recent fluoroquinolones, such as sparfloxacin. Again, exposure to sparfloxacin selects mutants that lead to greater resistance to gatifloxacin (Fukuda et al., 1998; Sanders, 2001).

In addition to mutations in genes encoding their targets, quinolone resistance can be achieved by the activity of resistance determinants that can be intrinsic (multidrug efflux pumps) or acquired [Qnr, QepA, OqxAB or AAC(6')-Ib-cr]. These resistance determinants are more proficient against the first generation of quinolones, and their activity against fluoroquinolones and later generations is less, but clinical resistance can be reached through overexpression (Garoff et al., 2018). In the case of Qnr, which shields the target of quinolones, there are mutational alleles presenting various degrees of protection against ciprofloxacin (Rodríguez-Martínez et al., 2009; Tavío et al., 2014); whether they act in parallel in all quinolones remains unclear. In addition, it has also been shown that the presence of transferable quinolone resistance determinants, often conferring non-clinically relevant phenotypes, favors the selection of mutations in other chromosomal targets that act cooperatively to increase MIC to quinolones (Cesaro et al., 2008; Li et al., 2019). However, it is clear that older quinolones (such as nalidixic acid) should efficiently select for QnrA1, QnrB1, QnrS1, AAC(6')-Ib-cr, and QepA (Hooper and Jacoby, 2016). Each of these genes might present different alleles, and the effect of each of these alleles in the resistance profile can vary. The study of environmental microbiomes has detected an AAC(6')-Ib-cr allele (the WY variant) that presented increased activity against gemifloxacin and was less active against ciprofloxacin compared with the “wild-type” -cr allele. Note that these alleles are unlikely to be the result of selection in clinics, given they have not yet been encountered in isolates from patients (Kim et al., 2018). Also, it has been shown for the first time that *in vitro* substitutions causing mutations in the structural elements of intrinsic multidrug efflux pumps can alter their substrate specificity profile within members of the same family of antibiotics (Blair et al., 2015). Amino acid substitution within the drug binding pocket of an efflux pump protein (AcrB) caused selection of relevant ciprofloxacin resistance in *Salmonella Typhimurium*, and in some cases, nalidixic acid resistance was increased more than for ciprofloxacin (allogeneous hyper-resistance). However, mutations in the pump SmeH of *S. maltophilia* (previously discussed) increased norfloxacin resistance without altering the susceptibility to nalidixic acid or ofloxacin (Blanco et al., 2019).

## Allogeneous Selection Within Aminoglycosides

Allogeneous selection appears to be rare or nonexistent in aminoglycosides. Despite prolonged use of members of this family of antibiotics, nothing comparable to the emergence of hundreds of mutant extended-spectrum β-lactamases under β-lactam exposure has occurred, and no mutant derivatives of aminoglycoside-modifying enzymes have been reported in clinical isolates (Toth et al., 2010). Among the more frequent inactivating enzymes are the AAC(6') enzymes, with three families that have been recognized by phylogenetic analysis. The possibility of the *aac(6')-Iaa* gene evolving to increased levels of resistance to gentamicin, tobramycin, kanamycin or amikacin and to acquire resistance to gentamicin was assessed by *in vitro* evolution experiments, which did not succeed in obtaining alleles with increased MICs (Salipante and Hall, 2003).

In addition to classical inactivating enzymes, several traits unrelated to classical antibiotic resistance determinants (most of them participating in pathways of bacterial metabolism) contribute to intrinsic resistance to aminoglycosides. Given their role in basic bacterial physiology, their activity should be similar to all aminoglycosides. Unexpectedly, in a study on *P. aeruginosa* seeking changes in MICs of four aminoglycosides by transposon-tagged insertion mutagenesis, the majority of mutants did not show changes in MICs for any of four studied aminoglycosides (streptomycin, kanamycin, tobramycin, amikacin), suggesting a certain degree of specificity of these “metabolism-derived resistance-traits” (Sanz-García et al., 2019). Apramycin-resistant mutants might have reduced MICs to streptomycin, kanamycin or neomycin; again, this is a possible case of antagonistic pleiotropy (Walton, 1978).

If evolution towards broader spectrum or higher enzymatic activity is very rare in aminoglycoside-resistance genes in Enterobacterales, which might be related with high fitness costs of these variants (Kramer and Matsumura, 2013), that is compensated by collecting several different genes. That is possibly the result of the high diversity and availability of these enzymes in nature, frequently with diverse phylogenetic origin (Salipante and Hall, 2003).

## Allogeneous Selection in Other Antibiotic Families: Glyco-Lipopeptides and Inhibitors of Folate Metabolism

Vancomycin and avoparcin select for *vanA* and *vanB* glycopeptide resistance determinants in enterococci. In the resistance process, there are two-component regulatory systems (VanR-VanS and VanRB-VanSB) that are inducers of the expression of resistance genes. Teicoplanin is a poor inducer (badly recognized); thus, strains with *vanA* or *vanB* might remain teicoplanin-susceptible. However, mutations in the genes involved in these regulatory systems might evolve teicoplanin resistance (Baptista et al., 1996). Vancomycin exposure might yield *Staphylococcus capitis* mutants with increased resistance to daptomycin (Butin et al., 2015). In *Staphylococcus aureus*, vancomycin exposure selects several mutations giving rise to a vancomycin-intermediate phenotype (VISA), some of which also reduce the effect of daptomycin. However, vancomycin exposure rarely selects for *mprF* mutations in reduced daptomycin-vancomycin susceptibility (Thitiananpakorn et al., 2020).

Daptomycin-resistant mutants have been detected in clinical coagulate-negative *Staphylococcus*. Here again, mutations influence the effect of a two-component regulator, WalKR (Jiang et al., 2019). As previously mentioned, daptomycin-resistant mutants based on the phospholipid flippase MprF emerges under daptomycin exposure (Ernst et al., 2018). In vancomycin-resistant *Enterococcus faecium*, mutations in both *liaFSR* and cardiolipin synthase (*cls*) genes presented a high level of resistance to daptomycin (Wang et al., 2018).

Allogenous selection has not been detected among mutants to antifolate inhibitors. Sulfonamide mutational resistance (mostly by alterations in dihydropteroate synthase) appeared to have no impact on the level of trimethoprim resistance, given the trimethoprim MICs for four different strains resistant to sulfonamides but susceptible to trimethoprim and which were transformed to trimethoprim resistance remained unchanged from their original MICs (Adrian and Klugman, 1997).

## Synergistic Mutational Pleiotropy in Resistance Within Members of Antibiotic Families: an Evolutionary Accelerator?

Allogenous selection results from a pleiotropic effect that can be considered the opposite (synergistic pleiotropy) of antagonistic pleiotropy within a family (“*A*”) of antibiotics, in which a mutational event increasing resistance to the *a1* antibiotic reduces resistance to another *a2* antibiotic. The typical case of antagonistic pleiotropy can be illustrated by mutations of classic TEM-1, TEM-2, SHV-1, or ROB-1 β-lactamases, which are extremely active on aminopenicillins. Mutations influencing the β-lactamase omega loop, which are found in oxyimino-cephalosporin-resistant variants, reduce enzymatic stability in these β-lactamases (Poirel et al., 2001). Classic β-lactamases are thus converted into ESBLs, but at the expense of drastically reducing hydrolytic efficiency toward aminopenicillins and first-generation cephalosporins (Raquet et al., 1994; Matagne and Frère, 1995; Galán et al., 2003). Similarly, acquisition of mutational resistance to inhibitors of β-lactamases reduces the hydrolyzing activity against aminopenicillins and ESBLs, including CTX-M enzymes (Ripoll et al., 2011). A similar case occurs with the development of new mutational variants of the β-lactamase (carbapenemase) KPC-1; KPC-2; or KPC-3 reduce carbapenem MICs but also affect the inhibitor capacity of avibactam. Avibactam, however, is not a member of the β-lactam family (Gaibani et al., 2018; Giddins et al., 2018). Of course, antibiotic antagonistic pleiotropy (also called collateral susceptibility, but which includes antibiotics from other families) tends to slow the evolution of resistance with use of multiple antibiotics of the same family.

Antibiotic synergistic pleiotropy within families could eventually accelerate the development of resistance, because of the simultaneous evolution of resistance traits. It has been shown that those additive interactions and epistatic interactions resulting from exposure to different cephalosporins increase the ability of a TEM enzyme to provide higher fitness to the host cell than any single cephalosporin (Mira et al., 2021). The predictability of resistance phenotypes resulting from mutations to different antibiotics (Knopp and Andersson, 2018) suggests that a similar approach could be applied to mutations within a single antibiotic family.

Synergistic pleiotropy has been proposed to explain the evolution of adaptive traits in plants and animals (Frachon et al., 2017) and should be more effective in simpler organisms, such as bacteria. There is a “cost of complexity” that results in complex organisms adapting more slowly than simple ones when using mutations of the same phenotypic size (Orr, 2000; Wagner et al., 2008; Rocabert et al., 2020).

## Practical Conclusions: Being Aware of Common Mistakes

Within a single-family *A* of antibiotics, when resistance to an *a1* antibiotic emerges, a new *a2* antibiotic active in *a1* resistant organisms is often developed and introduced in clinical practice. However, it is frequently not known (untested) whether the introduction of *a2* will select *a2* mutants with increased resistance (MIC) to *a1* in *a1*-susceptible or low-level resistance strains. Thus, the increase in the use of *a2* (because of the hypothetical prevention of *a1* resistance) will eventually help to reduce the overall effect of the older drug. On the other hand, improvements in MICs with the newest drugs of the same family do not necessarily preclude the “old” antibiotics from retaining some selective power for resistance to the newest agents. If this is true, the use of older members of the same family would facilitate the emergence of mutational resistance to the younger drugs of the family, which is frequently based on previously established resistance traits.

It could be mistaken to consider the “older drugs” that have been replaced in high-income countries by more advanced ones (generally much more expensive) of the same family as “accessible” (cheaper) antibiotics to be used in low-income countries. These drugs will probably select for “modern resistance traits,” inactivating the novel members of the family. This phenomenon could explain (together with lack of proper sanitation) the increase in emerging resistance traits such as NDM-1 β-lactamase in low-income countries (see above) among low-income countries to β-lactamases such as NDM-1 (see above). The same is true for certain “old antibiotics” used in livestock or agriculture.

The rapid worldwide propagation of certain multiresistant bacterial clones, such as *E. coli* ST131 harboring CTX-M-15, or in *Klebsiella* and *E. coli* clones harboring NDM-1, even in continents with scarce use of expensive extended-spectrum cephalosporins or carbapenems, could be due to local selection of these clones by old, inexpensive, widely used antibiotics such as aminopenicillins or first-generation cephalosporins (Walsh et al., 2005; Ghiglione et al., 2018) and their subsequent spread in poor sanitary conditions (Iskandar et al., 2020).

**Figure 2:**
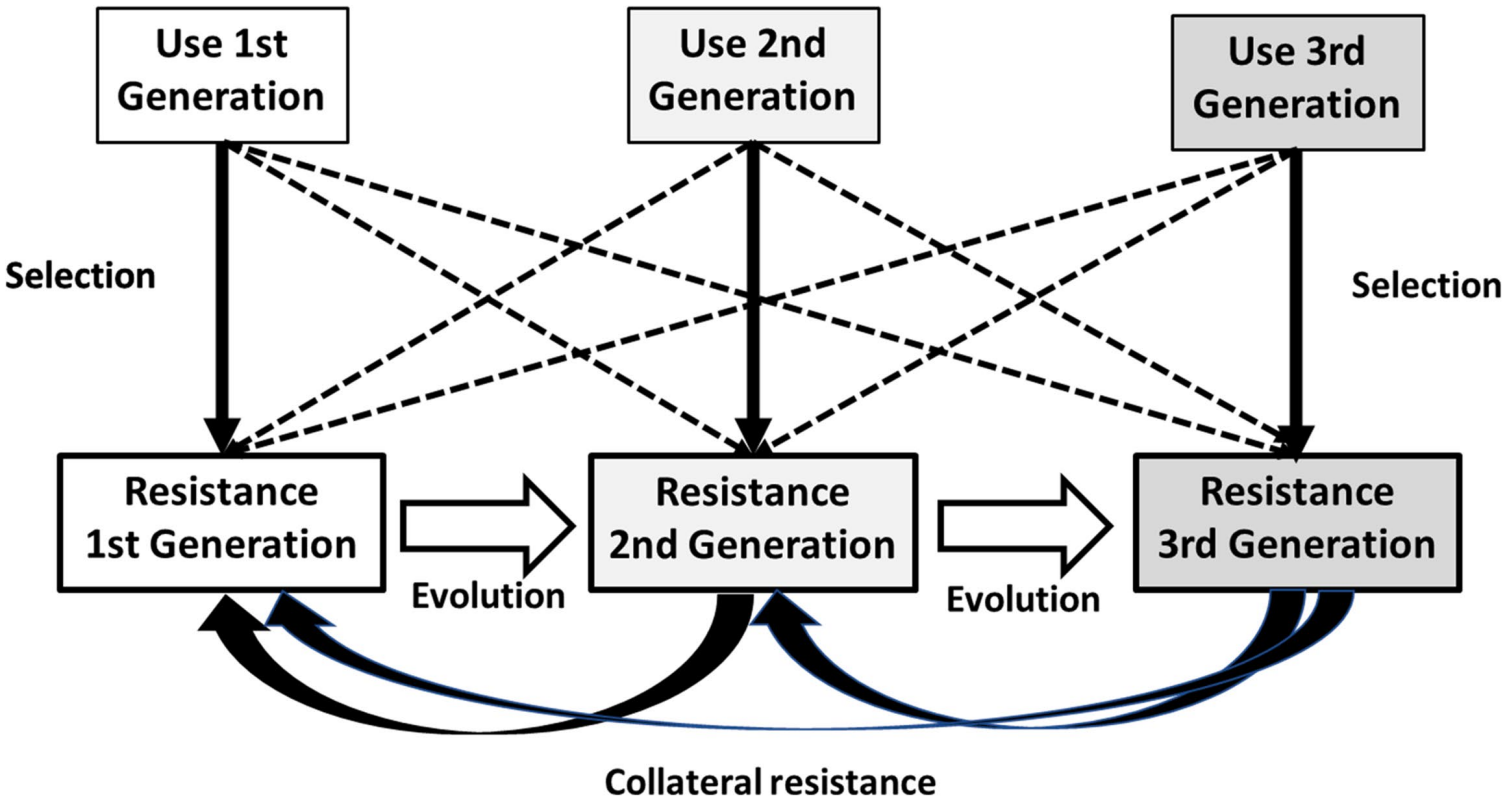
Key role of first-generation antibiotics within a family of drugs in maintaining and extending antibiotic resistance. Each generation of drugs preferentially selects resistance to the corresponding generation (vertical thick arrows) but might select for resistance to other generations (broken arrows). Quantitative expansion of resistance to first-generation drugs provides a wealth of genetic sequences from which evolution to resistance to second- and third generation drugs takes place (white horizontal arrows). Resistance to second- and third-generation antibiotics frequently inactivates (and then selects by collateral resistance, curved arrows) resistance to first-generation drugs, which is amplified by the constant use of all members of the family and is thus maintained as a growing source of variation and selection of resistance to the whole family.

Policies based on restricting the use of specific antibiotics as a response to increases in resistance should take into consideration of the role of old antibiotics in resistance selection, as the continued use of the “old” antibiotics of the same family could provide a powerful selection field for antibiotic-resistant mutants to the newest ones. The mixture of “old” and “new” antimicrobials of the same family, or even different types of “new” ones in a local setting is frequently synergistic for the evolution of antibiotic resistance. In addition, in recent times there has been a “vintage trend” to revive old antibiotics (Falagas et al., 2008; Theuretzbacher et al., 2015); however, we should be aware of the risks of such an approach. In Figure 2, we show the key role of first-generation antibiotics (“old ones”) within a family of drugs in maintaining and extending antibiotic resistance.

In terms of future research, it might be advisable to focus antimicrobial drug discovery more on the identification of new targets and new (unique) classes of antimicrobial agents than on the perpetual chemical exploitation of existing classic ones. Of course, that will not prevent the evolution of resistance to the members of these novel families, except if they are used very prudently or in combination therapy. In any case, the consideration of complex networks of collateral susceptibility (Imamovic and Sommer, 2013), and collateral resistance (allogenous selection) merits further exploration to reduce the dynamics of evolutionary paths and trajectories in antibiotic resistance (Baquero et al., 2021).

## Author Contributions

All authors listed have made a substantial, direct and intellectual contribution to the work, and approved it for publication.

## Funding

This work was supported (FB and TC) by Fundación Ramón Areces and the Instituto de Salud Carlos III (PI18/1942) and co-funded by the European Regional Development Fund (ERDF, ‘A way to achieve Europe’). Also supported by InGEMICS-CM (B2017/BMD-3691), funded by Comunidad de Madrid (Spain) and CIBER (CIBER in Epidemiology and Public Health, CIBERESP; CB06/02/0053), integrated in the Spanish 2013–2016 I+D+i State Plan and funded by Instituto de Salud Carlos III. ÂN is supported by national funds through FCT in the context of the transitional norm [DL57/2016/CP1346/CT0032].

## Conflict of Interest

The authors declare that the research was conducted in the absence of any commercial or financial relationships that could be construed as a potential conflict of interest.

## Publisher’s Note

All claims expressed in this article are solely those of the authors and do not necessarily represent those of their affiliated organizations, or those of the publisher, the editors and the reviewers. Any product that may be evaluated in this article, or claim that may be made by its manufacturer, is not guaranteed or endorsed by the publisher.

## Footnotes

^1^ http://bldb.eu/BLDB.php?prot=A#CTX-M
